# Association of Circulating COMP and YKL-40 as Markers of Metabolic Changes of Cartilage with Adipocytokines in Juvenile Idiopathic Arthritis

**DOI:** 10.3390/metabo10020061

**Published:** 2020-02-10

**Authors:** Katarzyna Winsz-Szczotka, Kornelia Kuźnik-Trocha, Anna Gruenpeter, Magdalena Wojdas, Klaudia Dąbkowska, Krystyna Olczyk

**Affiliations:** 1 Department of Clinical Chemistry and Laboratory Diagnostics, Faculty of Pharmaceutical Sciences in Sosnowiec, Medical University of Silesia, ul. Jedności 8, 41-200 Sosnowiec, Poland; kkuznik@sum.edu.pl (K.K.-T.); magdalena.bacia@gmail.com (M.W.); klaudia_092@vp.pl (K.D.); olczyk@sum.edu.pl (K.O.); 2 Department of Rheumatology, The John Paul II Pediatric Center in Sosnowiec, ul. Gabrieli Zapolskiej 3, 41-218 Sosnowiec, Poland; anna.gruenpeter@gmail.com

**Keywords:** juvenile idiopathic arthritis, cartilage degradation markers, cartilage oligomeric matrix protein, human cartilage glycoprotein 39, adipocytokines, leptin, adiponectin, resistin

## Abstract

The aim of this study was to evaluate the association of circulating cartilage oligomeric matrix protein (COMP) and human cartilage glycoprotein-39 (YKL-40) as markers of metabolic changes of cartilage, with leptin, adiponectin, and resistin in juvenile idiopathic arthritis (JIA) patients before and after treatment. A significant decrease of COMP and an increase of YKL-4 were found in blood of untreated patients. JIA treatment leading to clinical improvement resulted in normalization of COMP levels only. Concentrations of both markers in treated patients, while showing no clinical improvement, differed from those in controls and patients with remission. The leptin level decreased (*p* < 0.05) in untreated patients; however, concentrations of adiponectin and resistin increased (*p* < 0.05) as compared to controls. JIA treatment resulted in normalization of adipocytokine levels in remissive patients but not those with active JIA. Untreated patients showed a correlation between COMP and leptin, adiponectin, and body mass index (BMI) and between YKL-40 and leptin, adiponectin, BMI, C-reactive protein (CRP), and erythrocyte sedimentation rate (ESR). In inactive JIA, a correlation between YKL-40 and leptin was shown. Treated patients with an active JIA demonstrated a correlation between COMP and adiponectin and between YKL-40 and leptin, adiponectin, BMI, CRP, and ESR. The results of this work indicate that leptin and adiponectin but not resistin may be involved in the development and progression of joint dysfunction in JIA. Additionally, we suggest that YKL-40 may be a useful biomarker of disease activity and may be used to assess treatment towards remission, as compared to COMP.

## 1. Introduction

Juvenile idiopathic arthritis (JIA) is the most prevalent inflammatory disease of connective tissue in pediatric patients [1,2,3]. The progressive wear of articular cartilage in the course of JIA results in imbalance between biological resistance of the cartilage, its function, and the contact force. Such disorders are attributed to changes in homeostasis of the extracellular matrix (ECM) components of cartilage. Among the components, proteoglycans, including aggrecan, decorin, or biglycan, play a special role, maintaining the mechanical and immune properties of cartilage [1,2,3,4,5]. In our previous studies, we found that, in the course of JIA, there occurred a dysregulation of cartilaginous ECM remodeling, manifested by significant changes of the circulating markers of cartilage turnover, including total glycosaminoglycans (GAGs) and their particular types such as keratan sulphate, chondroitin sulphate, hyaluronic acid, as well as chondroitin sulphate 846 epitope [5,6,7,8].

Among ECM components, which are indicators of the cartilage breakdown, there are also listed components synthesized by chondrocytes, i.e., cartilage oligomeric matrix protein (COMP) as well as human cartilage glycoprotein 39 (YKL-40), that are so far not evaluated in JIA patients. The first one is a non-collagenous glycoprotein, binding to aggrecan; fibronectin; and collagen types I, II, and IX, which seem to play a role in the structure of fibrils and in maintenance of the collagen network [9,10]. Whereas YKL-40, a glycoprotein associated with inflammation and tissue remodeling, is produced by joint tissues and recognized as a candidate autoantigen in rheumatoid arthritis. It has been shown that YKL-40 binds to some important components in the cartilage extracellular matrix, i.e., proteoglycans and collagens, influencing their production and assembly [11,12,13,14]. Several authors have investigated the relationship between circulating COMP or YKL-40 and the condition of articular cartilage in adult patients with and without any rheumatic disease [9,10,11,12,13,14,15,16,17,18,19]. A correlation between the degree of articular cartilage degradation expressed by COMP level but not by YKL-40 and the adipose tissue content has been observed in the patients [15]. So far, the abovementioned correlation has not been evaluated in children with JIA. It should be pointed that the adipose tissue is a significant source of pro-inflammatory agents, also supporting the development of JIA. The numerous and complex functions of adipocytokines, including leptin, adiponectin, and resistin, secreted to the bloodstream result in relations between the adipose tissue, metabolic disorders, and autoimmune inflammatory diseases [15,20,21,22,23].

Given the abovementioned relations, the aim of our study was to evaluate COMP and YKL-40 concentrations in blood as potential markers of joint dysfunctions in patients with newly diagnosed JIA and in the same patients, both after clinical improvement, observed following an inflammation modifying therapy as well as in patients where the therapy prescribed did not result in remission.

Since metabolism of the cartilage ECM components is probably associated with hormonal activity of the adipose tissue, we decided to assess the relationship between serum levels of COMP and YKL-40 and some selected regulatory molecules, such as leptin, adiponectin, and resistin levels in patients with JIA. We have also evaluated the interactions between COMP, YKL-40, and the body mass index (BMI) as well as the inflammatory indicators, i.e., C-reactive protein (CRP) and erythrocyte sedimentation rate (ESR).

## 2. Results

The results are presented in Table 1.

### 2.1. The Concentration Changes of COMP

It was observed that serum concentration of COMP in children with untreated JIA was significantly lower (*p* < 0.05), i.e., by 28%, than that in healthy children. Furthermore, it was noted that the therapy modifying the course of disease and contributing to clinical improvement affected also a significant (*p* < 0.005) elevation of the assessed marker levels in the serum of these patients (inactive disease). Patients with inactive disease showed that concentrations of the assessed cartilage damage indicator normalized by pharmacotherapy. On the other hand, blood concentrations of COMP in patients insensitive to the therapy (active disease) remained significantly higher as compared to both concentrations in the controls (*p* < 0.05) and the treated remissive JIA patients (*p* < 0.05).

Analysis of the relations between COMP concentration and BMI as well as the inflammatory indicators which are routinely evaluated, i.e., CRP and ESR, revealed a significant relationship only with BMI in children with newly diagnosed and untreated JIA. The values were as follows: COMP with BMI (*r =* −0.49, *p* = 0.036), CRP (*r =* 0.15, *p* = 0.204), and ESR (*r =* −0.17, *p* = 0.132). We recorded insignificant relationships between COMP with BMI (*r =* 0.02, *p* = 0.350) and CRP (*r =* −0.20, *p* = 0.231) as well as COMP with ESR (*r =* −0.18, *p* = 0.184) in patients with JIA whose clinical condition had stabilized (inactive disease). Similarly, insignificant relationships between COMP with BMI (*r =* −0.18, *p* = 0.06) and CRP (*r =* 0.34, *p* = 0.089) as well as COMP with ESR (*r =* 0.09, *p* = 0.225) were observed in the treated patients with active JIA (active disease) (Table 2).

### 2.2. The Concentration Changes of YKL-40 

It was indicated that serum concentrations of YKL-40 in children with untreated JIA were significantly higher (*p* < 0.001), i.e., by 105%, as compared to blood concentrations in healthy children. Furthermore, it was observed that the therapy employed in JIA patients resulted in the significant decrease (*p* < 0.005) in serum YKL-40 levels only in JIA remissive children (inactive disease). Nevertheless, both groups of the treated JIA patients still showed markedly higher serum levels of the mentioned parameter, as compared to the controls. When compared to the control values, the mean increases in YKL-40 level in patients with inactive disease were higher by 19% (*p* < 0.05) and by 94% (*p* < 0.001) in patients with the active disease. Furthermore, blood concentrations of the evaluated marker in the treated JIA patients with no clinical improvement were significantly higher (*p* < 0.05), approximately by 62% as compared to serum YKL-40 concentrations in the treated remissive patients. Analysis of the relations between YKL-40 concentration and BMI, CRP, and ESR revealed a relationship between these parameters in patients with newly diagnosed and untreated JIA as well as in the treated patients with active disease. In the untreated patients, the following values were observed: YKL-40 with BMI (*r =* 0.50, *p* = 0.014), CRP (*r =* 0.68, *p* = 0.0001), and ESR (*r =* 0.55, *p* = 0.002). Those in the treated patients with active disease the relationships were as follows: YKL-40 with BMI (*r =* 0.42, *p* = 0.031), CRP (*r =* 0.60, *p* = 0.004), and ESR (*r =* 0.52, *p* = 0.009). We recorded insignificant relationships between YKL-40 and BMI (*r =* 0.34, *p* = 0.206), CRP (*r =* 0.26, *p* = 0.081), ESR (*r =* 0.06, *p* = 0.568) in patients with the treated JIA whose clinical condition had stabilized (inactive disease) (Table 2).

### 2.3. The Concentration Changes of Adipocytokines

As shown in Table 1, in JIA patients, concentrations of adipocytokines were characterized by different trends of alterations. Consequently, the quantitative assessment of leptin revealed a significantly lower (33%, *p* < 0.05) serum level of the parameter in untreated JIA patients, as compared to the controls. Anti-inflammatory treatment led to a significant increase (45%, *p* < 0.0005) in serum leptin concentration in patients with remission (inactive disease) vs. pretreatment status. In this group, the pharmacological therapy normalized concentrations of the assessed adipocytokine. On the other hand, leptinaemia in the treated patients with active JIA was comparable to the leptinaemia values observed before the treatment and significantly lower from those characteristic of the controls (38%, *p* < 0.001) and the treated remissive patients (36%, *p* < 0.05). 

It was also shown that inflammation, promoting development of JIA, at the same time led to significantly elevated levels of adiponectin and resistin. The untreated patients had higher by 15% (*p* < 0.05) levels of adiponectin and by 18% (*p* < 0.05) levels of resistin than the controls. Clinical improvement (inactive disease) was accompanied by a statistically significant decrease in adiponectin (11%, *p* < 0.005) and resistin (19%, *p* < 0.05), vs. the pretreatment status. Concentrations of both assessed adipocytokines in the blood of children with clinically stabilized disease were not statistically (*p* > 0.05) different from those in the serum of healthy children. Furthermore, blood adiponectin concentrations in the treated patients showing no clinical improvement (active disease) were statistically (*p* < 0.05) higher as compared to adiponectinaemia in the healthy children and the treated patients with inactive JIA, while serum resistin levels in the treated patients with the active disease corresponded (*p* > 0.05) to concentrations in the remaining evaluated groups.

### 2.4. The Correlations between Markers of Metabolic Changes of Cartilage and Adipocytokines

Correlation analysis results of which are presented in Table 2 revealed that untreated JIA patients showed a significant correlation between serum COMP levels with leptin (*r =* 0.48, *p* = 0.048) and adiponectin (*r =* −0.42, *p* = 0.024). No correlation was recorded between serum COMP level and resistin (*r =* −0.32, *p* = 0.102). On the other hand, in treated children with inactive JIA, we observed no significant correlation between serum COMP level with leptin (*r =* 0.06, *p* = 0.410), adiponectin (*r =* 0.004, *p* = 0.336), and resistin (*r =* −0.13, *p* = 0.256). We recorded insignificant relationships between COMP with leptin (*r =* 0.35, *p* = 0.326) and resistin (*r =* 0.25, *p* = 0.168) but significant relationships with adiponectin (*r =* −0.64, *p* = 0.01) in patients with the treated JIA whose clinical condition had not stabilized (active disease).

Furthermore, the correlation analysis (Table 2) revealed a significant correlation in the untreated JIA patients between serum YKL-40 with leptin (*r =* −0.49, *p* = 0.004) and adiponectin (*r =* 0.52, *p* = 0.01) levels. No correlation was recorded between serum YKL-40 level and resistin (*r =* 0.21, *p* = 0.211). We recorded significant relationships between YKL-40 and leptin (*r =* −0.36, *p* = 0.031) as well as insignificant relationships with adiponectin (*r =* 0.05, *p* = 0.464) and resistin (*r =* −0.06, *p* = 0.198) in patients with JIA whose clinical condition had stabilized (inactive disease). Furthermore, there was a significant correlation in the treated, active JIA patients between YKL-40 and lepitn (*r =* −0.56, *p* = 0.006) as well as adiponectin (*r =* 0.38, *p* = 0.026). No correlation was recorded between YKL-40 and resistin (*r =* 0.48, *p* = 0.265). No corresponding correlations were found in the controls.

## 3. Discussion

The role of adipocytokines was earlier believed to be associated with pathogenesis of nutritional disorders and the related changes of the body mass. However, due to their ability to model the immune-inflammatory response [22,23], the role of adipocytokines, including leptin, adiponectin, or resistin, in the development of JIA is very likely yet not fully understood. It has been proven that the inflammatory process accompanying JIA is frequently connected with a negative energy balance and the loss of body mass [24]. Such a loss was confirmed by our results. We have shown a significant decrease in BMI in children with active forms of JIA. The results correspond to observations reported by other authors [24,25,26]. 

The recorded changes in BMI in children with JIA are probably related to the stimulated activity of pro-inflammatory cytokines, including tumor necrosis factor-α (TNF-α), interleukin-1 (IL-1) and IL-6, and muscle atrophy referred to as “rheumatoid cachexia” [25,26]. The above-listed cytokines were proven to increase by the way of feedback the expression of the gene encoding anorexigenic leptin. Suppressing appetite, the secreted leptin may decrease the patients’ body mass. Moreover, leptin itself is able to induce synthesis of the abovementioned pro-inflammatory substances [20,21]. Although, the levels of leptin in the blood of untreated patients evaluated in the our study do not seem to confirm explicitly such a hypothesis, the described mechanism of body mass loss in the afflicted children is likely to occur in the preclinical stages of the disease. Perfetto et al. [24] also consider the body mass loss accompanying development of JIA to be the main cause of decreased leptinaemia in the patients. The quoted authors did not show any correlation between blood leptin level and JIA activity or the type of disease. Likewise, Elwakkad et al. [27] did not confirm the correlation between leptin level and the disease activity; however, the mentioned adipocytokine level was high in JIA patients [27]. 

Observed leptinaemia in patients with untreated JIA may result in additional changes within the immune system of the patients, contributing to the development of arthropathy. The discussed adipocytokine is a link between the immune response, especially the Th1-dependent one, and the adipose tissue content in the system body [20,21,28]. This relation is the effect of both direct influence of leptin on functions of the immunocompetent cells as well as the result of cross-reactions. The latter ones are associated, on the one hand, with structural homology of leptin relative to the family of helical cytokines, including IL-6, IL-11, or IL-12 and on the other hand with the similarity of leptin receptors to the structure of type I cytokine receptors, including receptors for IL-6 or leukocyte inhibitory factor [21,28]. Leptin receptors are known to be present on the surface of monocytes/macrophages, neutrophils, subpopulations of T and B cells, or NK cells [20,29]. As a result of stimulation of the abovementioned receptors, phagocytosis increases due to activation of phospholipase followed by the increase in synthesis and release of eicosanoids, reactive oxygen species (ROS), and the pro-inflammatory cytokines [15,20,28,29].

These mechanisms stimulated by leptin, which shape the immune response, seem to lead to the development of early preclinical stages of JIA. However, leptin locally secreted in the joints seems to especially contribute to manifestation of JIA [30,31]. Locally synthesized adipocytokines may have a greater role in regulation of cartilage homeostasis since its concentration in the synovial fluid is higher than in the corresponding sera of patients [26]. The influence of leptin on articular cartilage remodeling is a complex phenomenon. It is related, among other things, to the activation of inducible nitric oxide synthase, which synergically occurs with the effects of pro-inflammatory cytokines, mainly IL-1 or TNF-α [20,21,28]. Synthesized nitrogen oxide promotes the loss of chondrocyte phenotype and induces apoptosis of these cells. Moreover, leptin enhances activation of matrix metalloproteinases (MMPs) and ROS [15,20,30,31,32] and promotes degradation of the articular cartilage.

Association of adipocytokines with the articular cartilage changes in JIA children confirms a significant relationship of both leptinaemia and BMI with serum COMP and YKL-40 concentrations, as demonstrated in this study. In contrast to Bjørnhart et al. [33] and Urakami et al. [34] but similar to Lewander et al. [35], we did not show any correlation between COMP and the severity of the inflammatory process. Significantly lower concentrations of COMP in the pretreatment patients and those in which methotrexate therapy failed to bring any clinical improvement are likely to manifest extensive cartilage degradation occurring at the early, active stages of the disease. When the clinical symptoms of JIA occur, the synthesis processes of ECM components do not compensate for the extent of degradation of these compounds. That is why blood serum concentrations of COMP are reduced in children with the active forms of JIA. It has been suggested that COMP may be clinically applicable as a prospective pre-radiographic diagnostic indicator assisting development of treatment interventions at the early stage of cartilage defects [10,19].

Concentrations of YKL-40 in blood of JIA children, so far not studied by other researchers, are different from COMP levels. Observed in all the patients with JIA, the increase in serum concentrations of YKL-40, resulting from its increased production, is probably an expression of cartilage protection against the effects of the pro-inflammatory factors [13,14], including indirectly leptin. It is suggested that YKL-40 protects the extracellular matrix during tissue remodeling via suppression of different types of metalloproteinases [14,36]. This marker plays a role in proteoglycan synthesis; therefore, associated with ECM remodeling, it protects cells from undergoing apoptosis and functions as a growth factor for fibroblasts and chondrocytes [14]. YKL-40 is not released from the cartilage matrix during its degradation but rather produced by chondrocytes activated by an inflammatory stimulus [12]. It is assumed that circulating YKL-40 might reflect the activity of local and systemic inflammation. Several studies investigated the significance of YKL-40 in relation to CRP and ESR [11,37,38]. We also determined the association between YKL-40 levels in serum of the clinical active JIA patients and CRP and ESR. Our results suggest that YKL-40 may be a biomarker of the disease activity in JIA and may be used to assess treatment towards remission, as compared to COMP. The clinical utility of YKL-40 as a diagnostic or prognostic marker in JIA needs to be further clarified. This is especially important in children with clinically stabilized disease because we do not observe in them any normalization of this marker’s concentration.

Analyzing the role of the adipose tissue in pathogenesis of JIA, it should be remembered that it is not a homogeneous organ of endocrine secretion but a group of several similar glands secreting compounds which often have opposite effects on metabolism [20,28,30,31]. The differences in adiponectinaemia and resistinaemia, related to leptinaemia, demonstrated in the present study seem to confirm the above hypothesis. We showed significant high concentrations of adiponectin and resistin in the blood of children with newly diagnosed JIA as well as significantly higher levels of adiponectin in the blood of the treated patients without clinical improvement. Furthermore, we observed its significant correlation with COMP and YKL-40 only in the case of adiponectin. Although Gheita et al. [39] confirmed that resistin significantly correlated with the disease activity, our results seem to indicate that contribution of this adipocytokine to remodeling of the cartilage in JIA patients is less important than the role of leptin and adiponectin.

Views on the role of adiponectin in the development of JIA are not clear [40,41]. On the one hand, hyperadiponectinaemia seems to be a manifestation of the mechanism compensating for increased activity of the pro-inflammatory compounds [20,21]. It has been proven that adiponectin reduces severity of the inflammatory response, inhibiting the phagocytic activity of macrophages and decreasing the expression of the gene encoding TNF-α [42,43]. Chondroprotective activity of adiponectin seems to be confirmed by its high dependence on YKL-40, demonstrated in our study. Moreover, some authors associate the role of the discussed hormone with its protective influence on transformation of the ECM components of the cartilage [44]. It is considered that adiponectin stimulates reconstruction of the joints by the regulation of chondrocytes proliferation. The differentiation of chondrocytes enhances synthesis of both proteoglycans and collagen [45]. In addition, the mentioned adipocytokine limits the degradation processes of ECM components by inhibiting the expression of MMP-13 and by stimulating synthesis of the tissue inhibitors of metalloproteinases-2 [46]. Although the demonstrated significant relationships between adiponectinaemia and COMP and YKL-40 concentrations confirm the possibility of the abovementioned regulations in patients with JIA, we cannot ignore the relationship between this adipocytokine and the catabolic mechanisms. Adiponectin has been shown to increase the release of pro-inflammatory mediators, including IL-6, IL-8, MMP-3, or cyclooxygenase-2, by chondrocytes and synovial fibroblasts, which may aggravate the damage [47,48]. Local pro-inflammatory and erosive effects of adiponectin, like the leptin effects, may also result from its participation in modeling of vascular cell adhesion protein-h1 expression on the chondrocyte surface, which in combination with other molecules, facilitates adhesion of lymphocytes, monocytes, or eosinophils [49].

In the light of the above data, it is difficult to indicate the unequivocal, nevertheless obvious, role of adipocytokines in the chain of pathogenetic changes leading to articular cartilage dysfunction in the course of JIA. The observed relationships between adipocytokines and the joint turnover markers, especially YKL-40, as well as their concentrations in the group of treated patients with no clinical improvement indicate that treatment which modifies inflammation does not contribute simultaneously to total regeneration of the articular matrix components, pointing to the need for further treatment integrating also the appropriate diet. This is particularly important considering that pain experienced by the children during movement can contribute to the loss of the muscle mass, replacing it with the adipose tissue, which plays a significant role in regulation of the immuno-inflammatory processes underlying JIA development.

## 4. Materials and Methods

The study was carried out on the serum obtained from 96 children of both sexes (77 girls and 19 boys), aged 5–12 years, with the newly diagnosed JIA. All the patients were diagnosed and classified as oligoarthritis or polyarthritis (as per International League of Associations for Rheumatology criteria). Moderate disease activity was assessed in all patients (as per Juvenile Arthritis Disease Activity Score). Other forms of JIA as well as any other chronic and autoimmune diseases were considered as the exclusion criteria. Moreover, accuracy of the diagnosis was confirmed by laboratory tests, namely indicators of the inflammatory response, i.e., CRP (immunonephelometric assay) and ESR (Westergren method); by measuring the rheumatoid factor (latex enhanced immunoturbidimetric test); and by determining the titre of antinuclear antibodies (indirect immunofluorescence assay). BMI was calculated as body weight in kilograms divided by the square of height in meters (Table 3).

The tests that the study aimed at were repeated in 66 of the same group of patients in which the therapy modifying the course of the disease failed to improve the clinical condition, on average, 4.41 ± 0.86 months after the beginning of the therapy (in Table 1, Table 2 and Table 3, active disease) as well as in 30 patients after the therapy when the clinical improvement was observed, on average, 10.02 ± 0.61 months after the beginning of the therapy (in Table 1, Table 2 and Table 3, inactive disease). The clinical improvement was determined according to the American College of Rheumatology (ACR) Pediatric 30 criteria. The treatment with stable doses of NSAIDs (Non-Steroidal Anti-Inflammatory Drugs), oral glucocorticoids (at a maximum dose of 1 mg of a prednisone equivalent per kilogram per day, with gradual dose reduction), sulfasalazine (30 mg per square meter of body-surface area), and methotrexate (≤ 15 mg per square meter of body-surface area once a week) was prescribed. No other disease-modifying anti-rheumatic drugs or biologic agents were allowed.

The 66 treated patients, who did not show clinical improvement, were classified to another therapy.

The reference material comprised of blood samples collected from 45 healthy children (34 girls and 11 boys), with the age matching the JIA patients, i.e., before puberty, and assessed according to the Tanner scale. The healthy children not included in our study did not suffer from any diseases which required hospitalization and did not undergo any surgical procedures during the previous year. Furthermore, they had not been treated pharmacologically just before the studies began, and their results of routine laboratory tests, i.e., blood cell morphology, blood levels of cholesterol, glucose, and creatinine, were normal for this age group (Table 3).

The serum obtained from both the healthy individuals and JIA patients was divided into portions and stored at −80 °C until initiation of the study. Determination of a single parameter was completed within a day; consequently, the inter-assay variation was insignificant.

Informed consent was obtained from the teenage participants, aged under 16, according to the ethical guidelines of the Declaration of Helsinki. Furthermore, parents or legal guardians signed the informed consent on behalf of all underaged patients. The Local Ethics Committee of the Medical University of Silesia approved of the research protocol used in this study (KNW/0022/KB/81/15).

The COMP, YKL-40, leptin, adiponectin, and resistin levels were measured using blindly tested coded serum samples, in duplicate. The serum concentrations of COMP (R&D Systems, Minneapolis, MN, USA), YKL-40 (QUIDEL Corporation, Athens, OH, USA), and all tested adipocytokines (BioVendor Research and Diagnostic Products, Brno, the Czech Republic) were measured by ELISA, following the manufacturer’s protocol.

### Statistical Analysis

The underwent results were statistically analyzed with the use of STATISTICA software by StatSoft, Inc. (StatSoft, Cracow, Lesser Poland, Poland) (data analysis software system) version 12.0 (www.statsoft.com). The normality of distribution was verified by Shapiro–Wilk test. The clinical data as well as the levels of YKL-40 and adiponectin obtained from healthy individuals and JIA patients were expressed as mean values and standard deviation. Since the variables were normally distributed, the parametric Student’s *t*-test was used to evaluate differences between the untied variables. To compare the same parameters in each patient, the paired Student’s *t*-test was used before the treatment and following restoration of clinical improvement.

In the case of COMP, leptin, and resistin, the obtained data were expressed as medians and interquartile ranges. Since the variables were not normally distributed, the nonparametric Mann–Whitney U test was used to evaluate differences between the untied variables (the controls and patients before the treatment as well as the controls and the same group of patients after treatment). Since the variables were not normally distributed, evaluation of differences between the groups made use of the Wilcoxon test for the tied quantitative variables in the JIA patients. The Spearman’s rank correlation coefficient was employed for statistical analysis of correlations between two variables. The *p*-values below 0.05 were considered significant.

## 5. Conclusions

The results of this work indicate that leptin and adiponectin but not resistin may be involved in the development and progression of joint dysfunction in JIA. Additionally, we suggest that YKL-40 may be a useful biomarker of disease activity and may be used to assess treatment towards remission, as compared to COMP.

## Figures and Tables

**Table 1:** The distribution pattern of serum cartilage oligomeric matrix protein (COMP), human cartilage glycoprotein-39 (YKL-40), and adipocytokines (leptin, adiponectin, and resistin) in healthy controls and juvenile idiopathic arthritis (JIA) patients.

Parameter	Control Subjects (*n* = 45)	Untreated JIA Patients (*n* = 96)	JIA Patients after Treatment	
			Inactive Disease (*n* = 30)	Active Disease *n* = 66
COMP (*µg/mL*) YKL-40 (*µg/L*) ^*^	0.57 (0.39–0.68) 82.57 ± 19.41	0.41 (0.25–0.58) ^a^ 169.16 ± 41.25 ^b^	0.56 (0.37–0.77) ^c^ 98.56 ± 36.97 ^a,c^	0.42 (0.38–0.48) ^a,f^ 160.10 ± 54.88 ^b,f^
Leptin (*ng/mL*)	7.33 (4.43–11.84)	4.90 (3.52–7.90) ^a^	7.11 (4.74–10.10) ^d^	4.55 (1.44–5.50) ^b,f^
Adiponectin (*µg/mL*) ^*^	19.37 ± 6.25	22.25 ± 3.87 ^a^	19.88 ± 4.95 ^c^	26.48 ± 10.94 ^a,f^
Resistin (*ng/mL*)	4.17 (3.69–4.83)	4.91 (3.96–6.77) ^a^	3.97 (2.87–4.93) ^e^	4.41 (2.37–5.89)

Results are expressed as the medians (quartile 1–quartile 3); ^*^ Results are expressed as mean ± SD; COMP, cartilage oligomeric matrix protein; YKL-40, human cartilage glycoprotein 39; ^a^ *p* < 0.05, ^b^ *p* < 0.001 compared to control group; ^c^ *p* < 0.005, ^d^ *p* < 0.0005, ^e^ *p* < 0.05 compared to untreated JIA patients; ^f^ *p* < 0.05 compared to treated JIA patients (inactive disease).

**Table 2:** Correlation analysis between serum cartilage oligomeric matrix protein (COMP) and human cartilage glycoprotein-39 (YKL-40) with leptin, adiponectin, resistin, BMI, C-reactive protein (CRP), and erythrocyte sedimentation rate (ESR) levels.

Parameter	Leptin r (*p*)	Adiponectin r (*p*)	Resistin r (*p*)	BMI r (*p*)	CRP r (*p*)	ESR r (*p*)
**Serum COMP**						
Untreated JIA patients (*n* = 96)	0.48 (0.048)	−0.42 (0.024)	−0.32 (NS)	−0,49 (0.03)	0.15 (NS)	−0.17 (NS)
JIA patients after treatment						
inactive disease (*n* = 30)	0.06 (NS)	0.004 (NS)	−0.13 (NS)	0.02 (NS)	−0.20 (NS)	−0.18 (NS)
active disease (*n* = 66)	0.35 (NS)	−0.64 (0.01)	0.25 (NS)	−0,18 (NS)	0.34 (NS)	0.09 (NS)
**Serum YKL-40**						
Untreated JIA patients (*n* = 96)	−0.49 (0.004)	0.52 (0.01)	0.21 (NS)	0.50 (0.014)	0.68 (0.0001)	0.55 (0.002)
JIA patients after treatment						
inactive disease (*n* = 30)	−0.36 (0.031)	0.05 (NS)	−0.06 (NS)	0.34 (NS)	0.26 (NS)	0.06 (NS)
active disease (*n* = 66)	−0.56 (0.006)	0.38 (0.026)	0.48 (NS)	0.42 (0.031)	0.60 (0.004)	0.52 (0.009)

Results are expressed as the Spearman’s rank correlation coefficients; COMP, cartilage oligomeric matrix protein; YKL-40, human cartilage glycoprotein 39; BMI, body mass index; CRP, C-reactive protein; ESR, erythrocyte sedimentation rate; *p* < 0.05, statistically significant; NS, not statistically significant.

**Table 3:** Clinical characteristics of control subjects and JIA patients.

Parameter	Control Subjects (*n* = 45)	Untreated JIA Patients (*n* = 96)	JIA Patients after Treatment	
			Inactive Disease (*n* = 30)	Active Disease (*n* = 66)
Age (years)	8.25 ± 2.03	8.23 ± 3.48	8.71 ± 3.70	7.95 ± 2.51
Sex, female/male	34/11	77/19	19/11	58/8
JADAS-27	-	18 ± 8.66	4 ± 2.48 ^b^	15 ± 4.57
BMI (kg/m^2^)	18.34 ± 2.12	16.18 ± 2.14 ^a^	18.02 ± 3.55 ^b^	16.67 ± 3.12 ^a^
WBC (10^3^/l)	7.85 ± 2.34	14.66 ± 4.59 ^a^	6.45 ± 2.70	9,72 ± 2,18 ^b^
RBC (10^6^/l)	4.75 ± 0.32	4.26 ± 0.42	4.62 ± 0.36	4,02 ± 0,33 ^a^
Hb (g/dl)	14.08 ± 0.74	11.25 ± 1.78 ^a^	12.94 ± 1.53 ^a,b^	12,02 ± 1,27 ^a,b^
Ht (%)	40.68 ± 3.26	35.67 ± 3.52 ^a^	37.22 ± 7.51 ^a,b^	37,19 ± 3,57 ^a,b^
PLT (10^3^/l)	284.42 ± 68.22	398.26 ± 111.87 ^a^	359.26 ± 80.06 ^b^	344,32 ± 70,15
Total cholesterol (mM)	4.32 ± 0.84	4.69 ± 1.39 ^a^	4.27 ± 1.55 ^b^	4.48 ± 0.69
Glucose (mM)	4.21 ± 0.38	4.18 ± 1.26	4.44 ± 0.56 ^b^	4.51 ± 0.93 ^b^
Creatinine (µM)	61.42 ± 12.45	77.58 ± 9.21 ^a^	64.35 ± 14.57 ^a,b^	82.58 ± 1.11 ^a,b^
CRP (mg/l)	1.20 ± 1.39	19.66 ± 21.68 ^a^	3.57 ± 0.62 ^b^	12,47 ± 16,88 ^a,c^
ESR (mm/h)	9.22 ± 7.41	41.66 ± 22.04 ^a^	12.01 ± 5.15 ^b^	24,95 ± 15,89 ^a,c^
ANA	-	57% (positive)	57% (positive)	57% (positive)
RF	-	100% (negative)	100% (negative)	100% (negative)

Results are expressed as mean ± SD; ^a^ *p* < 0.05 compared to control group; ^b^ *p* < 0.05 compared to untreated JIA patients; ^c^ *p* < 0.05 compared to treated JIA patients (inactive disease); BMI, body mass index; WBC, white blood cell; RBC, red blood cell; Hb, hemoglobin; Ht, hematocrit; PLT, platelet; CRP, C-reactive protein; ESR, erythrocyte sedimentation rate; ANA, antinuclear antibodies; RF, rheumatoid factor.

## References

[1] Twilt M., Pradsgaard D., Spannow A.H., Horlyck A., Heuck C., Herlin T. (2017). Joint cartilage thickness and automated determination of bone age and bone health in juvenile idiopathic arthritis. Pediatr. Rheumatol..

[2] Mitra S., Samui P.P., Samanta M., Mondal R.K., Hazra A., Mandal K., Sabui T.K. (2019). Ultrasound detected changes in joint cartilage thickness in juvenile idiopathic arthritis. Int. J. Rheum. Dis..

[3] Winsz-Szczotka K., Mencner Ł., Olczyk K. (2016). Metabolism of glycosaminoglycans in the course of juvenile idiopathic arthritis. Postepy Hig. Med. Dosw..

[4] Sophia Fox A.J., Bedi A., Rodeo S.A. (2009). The basic science of articular cartilage: Structure, composition, and function. Sports Health.

[5] Winsz-Szczotka K., Kuźnik-Trocha K., Komosińska-Vassev K., Jura-Półtorak A., Olczyk K. (2016). Laboratory indicators of aggrecan turnover in juvenile idiopathic arthritis. Dis. Markers.

[6] Winsz-Szczotka K., Kuźnik-Trocha K., Komosińska-Vassev K., Wisowski G., Gruenpeter A., Lachór-Motyka I., Żegleń B., Lemski W., Olczyk K. (2015). Plasma and urinary glycosaminoglycans in the course of juvenile idiopathic arthritis. Biochem. Biophys. Res. Commun..

[7] Winsz-Szczotka K., Komosińska-Vassev K., Kuźnik-Trocha K., Siwiec A., Żegleń B., Olczyk K. (2015). Circulating keratan sulfate as a marker of metabolic changes of cartilage proteoglycan in juvenile idiopathic arthritis; influence of growth factors as well as proteolytic and prooxidative agents on aggrecan alterations. Clin. Chem. Lab. Med..

[8] Winsz-Szczotka K., Komosińska-Vassev K., Kuźnik-Trocha K., Gruenpeter A., Lachór-Motyka I., Olczyk K. (2014). Influence of proteolytic-antiproteolytic enzymes and prooxidative-antioxidative factors on proteoglycan alterations in children with juvenile idiopathic arthritis. Clin. Biochem..

[9] Georgiev T., Ivanova M., Kopchev A., Velikova T., Miloshov A., Kurteva E., Yuzeir K., Penkov M., Kabakchieva P., Rashkov R. (2017). Cartilage oligomeric protein, matrix metalloproteinase-3, and Coll2-1 as serum biomarkers in knee osteoarthritis: A cross-sectional study. Rheumatol. Int..

[10] Tseng S., Reddi A.H., Di Cesare P.E. (2009). Cartilage oligomeric matrix protein (COMP): A biomarker of arthritis. Biomark. Insights.

[11] Väänänen T., Vuolteenaho K., Kautiainen H., Nieminen R., Möttönen T., Hannonen P., Korpela M., Kauppi M.J., Laiho K., Kaipiainen-Seppänen O. (2017). Glycoprotein YKL-40: A potential biomarker of disease activity in rheumatoid arthritis during intensive treatment with csDMARDs and infliximab. Evidence from the randomised controlled NEO-RACo trial. PLoS ONE.

[12] Väänänen T., Koskinen A., Paukkeri E.L., Hämäläinen M., Moilanen T., Moilanen E., Vuolteenaho K. (2014). YKL-40 as a novel factor associated with inflammation and catabolic mechanisms in osteoarthritic joints. Mediat. Inflamm..

[13] Kazakova M.H., Batalov A.Z., Mateva N.G., Kolarov Z.G., Sarafian V.S. (2017). YKL-40 and cytokines—A new diagnostic constellation in rheumatoid arthritis?. Folia Med..

[14] Huang K., Wu L.D. (2009). YKL-40: A potential biomarker for osteoarthritis. J. Int. Med. Res..

[15] King L.K., Henneicke H., Seibel M.J., March L., Anandacoomarasmy A. (2015). Association of adipokines and joint biomarkers with cartilage-modifying effects of weight loss in obese subjects. Osteoarthr. Cartil..

[16] Sakthiswary R., Rajalingam S., Hussein H., Sridharan R., Asrul A.W. (2017). Cartilage oligomeric matrix protein (COMP) in rheumatoid arthritis and its correlation with sonographic knee cartilage thickness and disease activity. Clin. Rheumatol..

[17] Lorenzo P., Aspberg A., Saxne T., Önnerfjord P. (2017). Quantification of cartilage oligomeric matrix protein (COMP) and a COMP neoepitope in synovial fluid of patients with different joint disorders by novel automated assays. Osteoarthr. Cartil..

[18] Skiöldebrand E., Ekman S., Mattsson Hultén L., Svala E., Björkman K., Lindahl A., Lundqvist A., Önnerfjord P., Sihlbom C., Rüetschi U. (2017). Cartilage oligomeric matrix protein neoepitope in the synovial fluid of horses with acute lameness: A new biomarker for the early stages of osteoarthritis. Equine. Vet. J..

[19] Hoch J.M., Mattacola C.G., Medina McKeon J.M., Howard J.S., Lattermann C. (2011). Serum cartilage oligomeric matrix protein (sCOMP) is elevated in patients with knee osteoarthritis: A systematic review and meta-analysis. Osteoarthr. Cartil..

[20] Mancuso P. (2016). The role of adipokines in chronic inflammation. Immunotargets Ther..

[21] Azamar-Llamas D., Hernández-Molina G., Ramos-Ávalos B., Furuzawa-Carballeda J. (2017). Adipokine contribution to the pathogenesis of osteoarthritis. Mediat. Inflamm..

[22] Coles C.A. (2016). Adipokines in healthy skeletal muscle and metabolic disease. Adv. Exp. Med. Biol..

[23] Booth A., Magnuson A., Fouts J., Foster M.T. (2016). Adipose tissue: An endocrine organ playing a role in metabolic regulation. Horm. Mol. Biol. Clin. Investig..

[24] Perfetto F., Tarquini R., Simonini G., Bindi G., Mancuso F., Guiducci S., Matucci-Cerinic M., Falcini F. (2005). Circulating leptin levels in juvenile idiopathic arthritis: A marker of nutritional status?. Ann. Rheum. Dis..

[25] Maciejewska-Paszek I., Grochowska-Niedworok E., Siwiec A., Gruenpeter A., Dul L., Irzyniec T. (2017). Influence of etanercept on leptin and ghrelin secretion in children with juvenile idiopathic arthritis. J. Int. Med. Res..

[26] Brzeska A., Brózik H., Lipińska J., Stańczyk J., Smolewska E. (2010). Leptin concentration in serum and synovial fluid of children with juvenile idiopathic arthritis. Reumatologia.

[27] Elwakkad A.S., Said R.N., Muhammad S.I., Saleh M.T., Elhamshary A. (2007). Role for leptin and prolactin in human juvenile rheumatic diseases. Pak. J. Biol. Sci..

[28] Tian G., Liang J.N., Wang Z.Y., Zhou D. (2014). Emerging role of leptin in rheumatoid arthritis. Clin. Exp. Immunol..

[29] Fernández-Riejos P., Najib S., Santos-Alvarez J., Martín-Romero C., Pérez-Pérez A., González-Yanes C., Sánchez-Margalet V. (2010). Role of leptin in the activation of immune cells. Mediat. Inflamm..

[30] Scotece M., Mobasheri A. (2015). Leptin in osteoarthritis: Focus on articular cartilage and chondrocytes. Life Sci..

[31] Hui W., Litherland G.J., Elias M.S., Kitson G.I., Cawston T.E., Rowan A.D., Young D.A. (2012). Leptin produced by joint white adipose tissue induces cartilage degradation via upregulation and activation of matrix metalloproteinases. Ann. Rheum. Dis..

[32] Koskinen A., Vuolteenaho K., Nieminen R., Moilanen T., Moilanen E. (2011). Leptin enhances MMP-1, MMP-3 and MMP-13 production in human osteoarthritic cartilage and correlates with MMP-1 and MMP-3 in synovial fluid from OA patients. Clin. Exp. Rheumatol..

[33] Bjørnhart B., Juul A., Nielsen S., Zak M., Svenningsen P., Müller K. (2009). Cartilage oligomeric matrix protein in patients with juvenile idiopathic arthritis: Relation to growth and disease activity. J. Rheumatol..

[34] Urakami T., Manki A., Inoue T., Oda M., Tanaka H., Morishima T. (2006). Clinical significance of decreased serum concentration of cartilage oligomeric matrix protein in systemic juvenile idiopathic arthritis. J. Rheumatol..

[35] Lewander P., Dahle C., Larsson B., Wetterö J., Skogh T. (2017). Circulating cartilage oligomeric matrix protein in juvenile idiopathic arthritis. Scand. J. Rheumatol..

[36] Ling H., Recklies A.D. (2004). The chitinase 3-like protein human cartilage glycoprotein 39 inhibits cellular responses to the inflammatory cytokines interleukin-1 and tumour necrosis factor-alpha. Biochem. J..

[37] Kazakova M., Batalov A., Deneva T., Mateva N., Kolarov Z., Sarafian V. (2013). Relationship between sonographic parameters and YKL-40 levels in rheumatoid arthritis. Rheumatol. Int..

[38] Baran A., Myśliwiec H., Szterling-Jaworowska M., Kiluk P., Świderska M., Flisiak I. (2018). Serum YKL-40 as a potential biomarker of inflammation in psoriasis. J. Dermatolog. Treat.

[39] Gheita T.A., El-Gazzar I.I., El Shazly R.I., El-Din A.M., Abdel-Rasheed E., Bassyouni R.H. (2013). Elevated serum resistin in juvenile idiopathic arthritis: Relation to categories and disease activity. J. Clin. Immunol..

[40] Markula-Patjas K., Valta H., Pekkinen M., Andersson S., Aalto K., Lahdenne P., Viljakainen H., Mäkitie O. (2015). Body composition and adipokines in patients with juvenile idiopathic arthritis and systemic glucocorticoids. Clin. Exp. Rheumatol..

[41] Ilisson J., Zagura M., Zilmer K., Salum E., Heilman K., Piir A., Tillmann V., Kals J., Zilmer M., Pruunsild C. (2015). Increased carotid artery intima-media thickness and myeloperoxidase level in children with newly diagnosed juvenile idiopathic arthritis. Arthritis Res. Ther..

[42] Liu D., Luo S., Li Z. (2015). Multifaceted roles of adiponectin in rheumatoid arthritis. Int. Immunopharmacol..

[43] Olczyk-Wrochna K., Wisłowska M. (2008). Adiponectin in rheumatoid arthritis. Reumatologia.

[44] Huang C.Y., Lee C.Y., Chen M.Y., Tsai H.C., Hsu H.C., Tang C.H. (2010). Adiponectin increases BMP-2 expression in osteoblasts via AdipoR receptor signaling pathway. J. Cell. Physiol..

[45] Challa T.D., Rais Y., Ornan E.M. (2010). Effect of adiponectin on ATDC5 proliferation, differentiation and signaling pathways. Mol. Cell. Endocrinol..

[46] Chen T.H., Chen L., Hsieh M.S., Chang C.P., Chou D.T., Tsai S.H. (2006). Evidence for a protective role for adiponectin in osteoarthritis. Biochim. Biophys. Acta.

[47] Li B.T., Zhang F.Z., Xu T.S., Ding R., Li P. (2015). Increasing production of matrix metalloproteinases, tumor necrosis factor-α, vascular endothelial growth factor and prostaglandin E2 in rheumatoid arthritis synovial fibroblasts by different adiponectin isoforms in a concentration-dependent manner. Cell. Mol. Biol..

[48] Kusunoki N., Kitahara K., Kojima F., Tanaka N., Kaneko K., Endo H., Suguro T., Kawai S. (2010). Adiponectin stimulates prostaglandin E(2) production in rheumatoid arthritis synovial fibroblasts. Arthritis Rheum..

[49] Conde J., Scotece M., López V., Gómez R., Lago F., Pino J., Gómez-Reino J.J., Gualillo O. (2012). Adiponectin and leptin induce VCAM-1 expression in human and murine chondrocytes. PLoS ONE.

